# The Role of Artificial Intelligence in Shaping the Doctor–Patient Relationship: A Narrative Review

**DOI:** 10.3390/healthcare14040481

**Published:** 2026-02-13

**Authors:** Emanuele Maria Merlo, Giorgio Sparacino, Orlando Silvestro, Maria Laura Giacobello, Alessandro Meduri, Marco Casciaro, Sebastiano Gangemi, Gabriella Martino

**Affiliations:** 1 Department of Biomedical and Dental Sciences and Morphofunctional Imaging, University of Messina, 98124 Messina, Italy; 2 Course Degree in Medicine and Surgery, Department of Adult and Childhood Human Pathology “Gaetano Barresi”, University of Messina, 98124 Messina, Italy; giorgio.sparacino@studenti.unime.it; 3 Department of Health Sciences, University Magna Graecia of Catanzaro, 88100 Catanzaro, Italy; orlando.silvestro@unicz.it; 4 Department of Ancient and Modern Civilizations, University of Messina, 98124 Messina, Italy; maria.giacobello@unime.it; 5 Ophthalmology Clinic, Department of Biomedical and Dental Sciences and Morphofunctional Imaging, University of Messina, 98124 Messina, Italy; alessandro.meduri@unime.it; 6 Department of Clinical and Experimental Medicine, University of Messina, 98124 Messina, Italy; mcasciaro@unime.it (M.C.); sebastiano.gangemi@unime.it (S.G.); gabriella.martino@unime.it (G.M.)

**Keywords:** artificial intelligence, AI, clinical psychology, doctor–patient relationship, evidence, human–computer interaction, internal medicine

## Abstract

**Highlights:**

**What are the main findings?**

- There is no universally accepted definition of Artificial Intelligence (AI) in medicine, despite its growing use in healthcare settings.
- Key challenges in the adoption of AI in clinical practice include diagnostic and therapeutic accuracy, data privacy, transparency, regulatory frameworks, cybersecurity, and ethical concerns.

**What are the implications of the main findings?**

- The integration of AI has the potential to influence the doctor–patient relationship, which remains a central element of clinical practice.
- Patients, medical doctors, and students generally view AI as a supportive tool rather than a replacement for human care, favoring its integration with traditional medical practice.

**Abstract:**

The doctor–patient relationship is a central factor in healthcare delivery. Artificial Intelligence (AI) represents an emerging technological frontier whose implications remain to be fully clarified. Evidence-based studies provide reliable analyses of effects and offer a deeper understanding of both limits and benefits. This narrative review aimed to explore the role of AI in modern clinical practice, with particular reference to its effects on the doctor–patient relationship. Scopus and Web of Science databases were searched between 1 and 10 December 2025 to identify suitable studies. Inclusion criteria comprised English-language articles published in the last 10 years, with a direct focus on the doctor–patient relationship and exclusively employing empirical research designs. A total of 21 studies published between 2021 and 2025 were identified as eligible. The most common AI applications were conceptual systems discussed at a perceptual level (thirteen studies), followed by simulated AI decision-making scenarios (two studies). Implemented AI applications were less frequent and mainly included AI-based clinical decision support systems, administrative and documentation-focused tools, and a small number of conversational or relational AI applications (six studies in total). These studies focused on patients, healthcare professionals, and medical students preparing for future clinical roles. Results highlighted generally positive patient attitudes toward AI, often mediated by educational level, technological familiarity, and risk awareness. Among healthcare professionals, positive attitudes also emerged, although concerns regarding epistemic and professional values were noted. Greater involvement of clinicians in its development was consistently recommended. Findings from academic samples aligned with those of patients and clinicians, showing that integrating AI with traditional clinical practices was consistently preferred. Empathy, compassion, effective communication, accuracy, ethics, and trust were highlighted as fundamental values essential for mitigating risks. These elements are fundamental to the effective implementation of technologies aimed at improving clinical practice, while an integrative perspective is needed to safeguard the doctor–patient relationship. Overall, the use of AI in medical practice emerged as promising. Further studies should strengthen the empirical basis of the field to support an evidence-based approach to AI integration in healthcare.

## 1. Introduction

Artificial Intelligence (AI) can be defined as the imitation of human intelligence by computers [1]. However, there is still no clear and universally accepted definition. Despite ongoing research into human cognitive abilities, strict definitions attempt to portray human functions to compare them with AI [2].

As a technology enabling machines to imitate complex human functions, AI must always be grounded in a solid scientific foundation. Following ethological approaches, intelligence is linked to adaptation and defined as the disposition to produce adaptive behaviour in response to environmental conditions and internal functioning [3]. Accordingly, several studies have compared AI with human intelligence, highlighting its limitations and conceptual boundaries [4,5].

From a historical perspective, AI has evolved through distinct developmental stages, including Artificial Narrow Intelligence (ANI), Artificial General Intelligence (AGI), and Artificial Super Intelligence (ASI) [6]. ANI refers to systems designed to perform specific tasks, while AGI denotes a flexible and adaptive intelligence capable of managing a wide range of cognitive activities. ASI, by contrast, is designed to exceed human capabilities in speed, quality, and decision-making [6,7,8].

The evolution of AI has generated new challenges and viewpoints, as well as limitations and ethical concerns. Its applications span multiple scientific fields [9]. Among them, medicine and the health sciences represent one of the most prominent frontiers [10,11,12,13].

Hirani and colleagues [14] recently suggested that the first use of AI in medicine dates to the 1960s, with the earliest conceptual questions arising in the 1950s. This long history has raised awareness of potential limitations, risks, and both general and specific concerns, such as excessive dependence on algorithms reducing human competence, clinical autonomy, and relational bonds. In this contest, several studies have emphasised the importance of addressing key issues when introducing AI into medical practice. These include the risks of unsustainable, groundless, or inappropriate human replacement [15,16,17], challenges related to diagnostic and therapeutic accuracy [18,19], and concerns about data privacy, transparency, regulation, and cybersecurity [20,21,22].

Additional challenges include automation bias, barriers to technological development, privacy and security concerns, and broader ethical implications [23,24,25,26,27]. These issues should be carefully considered.

Conversely, the use of AI represents a tangible advancement in medicine [28]. Its positive role and impact have been recognised in fields such as personalised treatment plans [29,30,31,32], early disease detection [33,34,35,36,37], improved trial efficiency [38,39,40], enhanced patient care and engagement [41,42,43,44], and error reduction [45,46]. Recent analyses of Artificial Intelligence applications across common clinical medicine domains highlight that even when AI improves efficiency and patient safety, its real-world value in clinical practice depends on transparent and ethically governed implementation with appropriate human oversight, factors that directly influence patient trust and the quality of the doctor–patient relationship [47].

AI has also demonstrated potential to reduce healthcare workers’ workloads and risk of burnout [48,49]. Patients and healthcare professionals represent the primary stakeholders in this context, and the resulting benefits suggest that AI can significantly advance care for both groups. The relationship between these parties is particularly relevant in healthcare.

The doctor–patient relationship plays a fundamental role in clinical practice. According to Oh Nelson [50], it can be considered one of the most essential components of healthcare. Its history, value, and impact can be traced across a wide historical period, from ancient practices to contemporary and emerging perspectives [51,52,53,54]. According to several studies, it can be defined in terms of the physician’s complex communication and interpersonal skills [52,55]. Evidence has highlighted the advantages associated with a person-centred gold-standard doctor–patient relationship, including improved satisfaction, staff retention, and reduced malpractice [56,57]. As a collaborative, trust-based bond between the healthcare professional and the patient, it is characterised by respect, empathy, fiduciary duty, honesty, shared decision-making, communication, and ethical foundations [58,59]. In this context, the introduction of new technologies into the relationship represents a new frontier [60].

Many factors can influence the clinical relationship [61], with potential implications for both parties involved. For healthcare professionals, psychological dynamics related to workloads, perceived quality of work, training received, and the nature of specific pathologies play a central role [62,63,64,65,66,67]. For patients, variables such as quality of life, disease type and chronicity, education level, and sociodemographic status are fundamentally important [68,69,70,71,72,73,74,75].

Given these complex dynamics, the introduction of technological tools can act as both a protective and a risk factor for the doctor–patient relationship [76,77,78,79,80]. For instance, such tools may support clinical decision-making, reduce administrative burden, and free time for patient interaction, while, at the same time, potentially undermining relational aspects of care through reduced human contact, over-reliance on algorithmic outputs, or concerns related to trust, transparency, and responsibility. Recent technological advances, such as AI, have brought this close relationship into sharp focus, making it a subject of considerable scientific interest. The influence of AI on the doctor–patient relationship is now an important scientific focus [56]. Empathy and compassion are widely recognised as foundational values in this context [81,82].

Nevertheless, although interest in this phenomenon is high, many contributions remain general or thematic [83,84,85]. While such reflections offer an essential foundation, empirical studies provide critical evidence through observation and experimentation.

This review aims to provide a narrative examination of empirical studies on the role of AI in the main domains of the doctor–patient relationship—namely, trust, communication, empathy, ethics, and technological advancement—to identify the primary relational outcomes associated with AI use in healthcare practice and offer a comprehensive understanding of the current state of knowledge and complexities introduced by AI.

## 2. Materials and Methods

The present review offers a rigorous synthesis of evidence on the influence of AI use with respect to the doctor–patient relationship. In line with SANRA indications [86] for justifying the research relevance, referencing the state of the art, and stating the concrete aims, the adopted approach enabled the identification and synthesis of relevant empirical studies. The searches started on 1 December 2025 ending on 10 December 2025.

Original empirical studies were identified through searches in the Scopus and Web of Science databases. The following keywords adapted for each database were used: (“artificial intelligence” OR “AI”) AND (“evidence” OR “effectiveness”) AND (“doctor patient relationship” OR “doctor patient relation” OR “doctor-patient relationship” OR “doctor-patient relation” OR “physician patient relationship” OR “physician patient relation” OR “physician-patient relationship” OR “physician-patient relation”).

Studies were included if they met the following criteria: published within the last 10 years, written in English, directly addressing the doctor–patient relationship, and employing empirical research methods. Observational quantitative–qualitative cross-sectional studies, prospective studies, trials, and interventional studies were considered as eligible. Only primary research studies explicitly referring to the doctor–patient relationship were analysed. Reviews, book chapters, case reports, and conference abstracts were excluded from the analysis. Two reviewers independently screened records, and any discrepancies were discussed to reach agreement. The selected studies were then analysed in depth to synthesise the emerging findings and clustered according to the main thematic domains. The narrative approach employed provides an interpretative and conceptual synthesis, aimed at enhancing understanding of AI’s role within clinical relationships.

## 3. Results

After retrieving the initial set of articles from Scopus (207) and Web of Science (205), for a total of 412 records, duplicates (6) were removed. The remaining records were then screened based on title and abstract.

**Figure 1:**
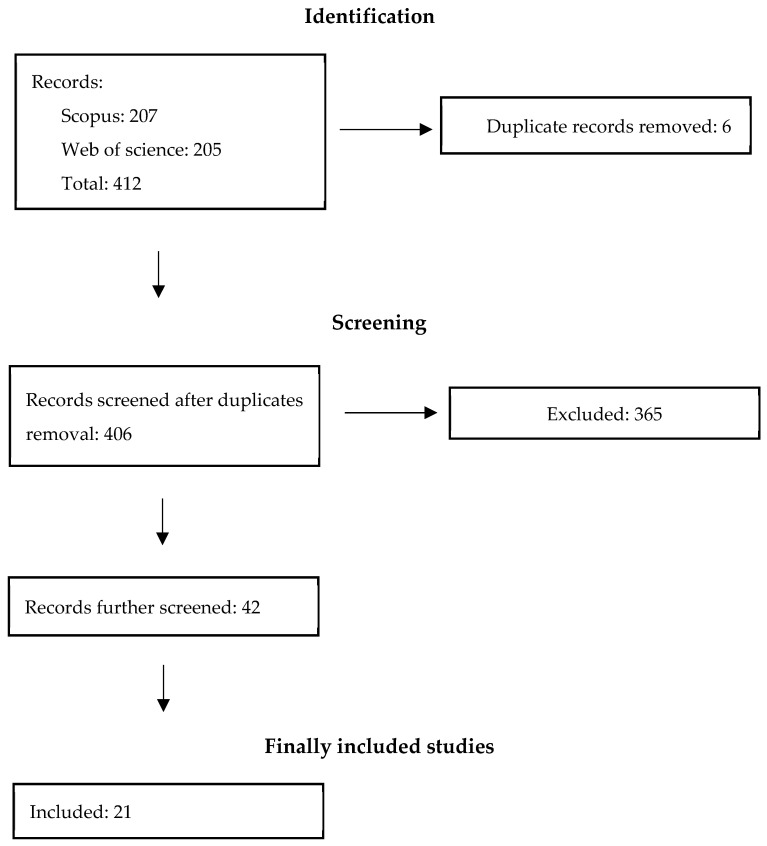
Search and selection diagram.

Of the 42 articles that passed this screening process, 21 full-text records met all the predefined inclusion criteria and were selected for inclusion in this narrative review and are presented in Table 1 and Figure 1. The results are presented through a structured narrative synthesis, with paragraphs organised according to study characteristics, patients’ and physicians’ perspectives, and key content domains directly related to AI use.

### 3.1. Main Characteristics of the Included Studies

Of the 21 finally included studies, according to design differences, 15 reported quantitative analyses [87,89,91,92,93,94,97,98,99,100,101,102,104,106,107], while 6 studies were qualitative [88,90,95,96,103,105]. In total, 10 studies adopted a cross-sectional quantitative design [87,89,91,92,94,99,101,104,106,107], 6 studies a qualitative cross-sectional design [88,90,95,96,103,105], three were prospective studies [93,98,102], one was a pilot randomised controlled trial [97], and one was an interventional study [100]. One study was conducted across Austria and Germany [101], three studies in China [94,104,107], one in Germany [103], one across Germany and Netherlands [92], one across Germany and USA [102], one in India [100], one in Italy [95], one in Japan [105], one in Pakistan [106], one in Portugal [99], one in Saudi Arabia [87], one across Switzerland, Germany, United Kingdom [90], one in Taiwan [93], two in the United Arab Emirates [88,96], and four in the USA [89,91,97,98]. All included studies were published between 2021 and 2025.

### 3.2. AI Use in Patients’ Perspective

Twelve of the twenty-one included studies examined patients’ experiences, expectations, attitudes, opinions, and outcomes related to AI use within the context of clinical practice. These studies comprised a heterogeneous range of designs, including cross-sectional quantitative surveys (the majority), qualitative interview-based studies, and a smaller subset of prospective studies, trials, and experimental or simulated scenarios [88,90,91,92,93,97,98,101,102,103,104,107].

Qualitative studies allowed the emergence of critical themes concerning AI use within the context of the doctor–patient relationship. Specifically, themes such as trust, potential distrust, and privacy concerns were identified as key dimensions [101]. Moreover, specific concerns emerged in relation to psychiatric practice, where empathy and trust were described as the fundamental basis of clinical relationships. Other issues included the risk of creating new relational barriers while neglecting existing problems [90].

Although a generally positive attitude toward AI emerged, aspects such as role perception, professional identity, and potential benefits should always be carefully considered. Schneider and colleagues [103] described AI integration as a new challenge, emphasising that its impact on the doctor–patient relationship should not erode shared decision-making processes. However, their study also revealed a significant limitation: patients’ level of information, knowledge, and understanding about AI plays a crucial role in shaping their opinions. Lack of adequate information can strongly influence attitudes and perceptions.

According to Alkaabi and colleagues [88], patients demonstrated positive attitudes toward integration processes that emphasise collaboration between doctors and AI, avoiding risks associated with exclusive reliance on AI systems. Analysing patients’ perspectives and experiences with AI use is inherently complex, and qualitative findings should ideally be compared with quantitative data to achieve a comprehensive understanding.

In line with these points, quantitative research also highlighted both concerns and perceived benefits. Studies reported worries that included the preservation of human contact and the doctor–patient relationship [91], increased costs [92], a preference for human interactions, and reduced satisfaction with care [104,107]. Conversely, positive attitudes were associated with higher education levels [91], the expressed need to anthropomorphise AI systems [107], increased diagnostic confidence, and reduced consultation times [92]. While overall attitudes were generally positive, the identification of specific limitations remains essential to address concerns and guide responsible implementation. From both qualitative and quantitative perspectives, it is critical to recognise both the positive potential and the challenges that must be addressed.

Prospective studies and trials offered additional insights. Owens and colleagues [98] reported that AI use for documentation and administrative tasks could support relational aspects of care, with no significant differences observed over time between AI-supported and traditional practices. AI was also represented improving adherence to physical therapies and strengthen social bonds [97]. Furthermore, empathy, trust, and the processing of affective responses during medical sessions were shown to be enhanced through AI, enabling the assessment of behaviours that could impact the clinician–patient relationship [93]. Even in these contexts, patients’ information, knowledge, and familiarity with technology significantly influenced their attitudes toward AI [102]. Overall, the integration of AI alongside traditional clinical practices was generally preferred, as it was seen as preserving the doctor–patient relationship.

### 3.3. AI Use in Doctors’ Perspective

According to the results, the included studies presented heterogeneous designs, comprising both quantitative and qualitative approaches, including cross-sectional surveys and interview-based qualitative studies, with a smaller number of mixed or prospective designs [87,88,89,90,94,95,96,99,105].

With reference to qualitative findings, results suggest that integrating AI with traditional clinical practices represents an important advancement [88]. Positive attitudes emerged, including perceived improvements in practice, enhanced decision-making, and favourable expectations [88,90,95,105].

However, potential risks were also identified. According to Amann and colleagues [90], new issues related specifically to human contact may arise from AI use. In line with this, Lombi & Rossero [95] suggested that although AI might not directly affect decision-making processes, it could pose threats to professional and epistemic status. Mansour and colleagues [96] highlighted that despite clear advantages, there are realistic risks of data misinterpretation and patient misunderstanding.

Consistent with the findings of Alkaabi and colleagues [88], doctors also emphasised that cultural and infrastructural challenges could represent concrete barriers, even though advantages were noted in terms of large-scale data processing [105]. Moreover, patients’ education, awareness, and information about AI emerged as key concerns, alongside suggestions to ensure doctors’ involvement in AI development phases [96].

Considering both the concerns and advantages identified through qualitative methods provides a solid foundation for future action.

On the other hand, quantitative results largely confirmed many of these concerns. According to several studies, factors such as sex, professional category, years of experience, low alignment between clinicians and stakeholders, risk of misdiagnosis and mistreatment, and algorithmic bias were identified as major issues for doctors [87,89,94].

Nevertheless, Al Fadeel and colleagues [87] reported that 64% of doctors expressed optimism about AI and demonstrated positive attitudes. Identified risks—including potential AI failure and the erosion of the doctor–patient relationship—must be addressed, for example, through strengthened collaboration between clinicians and stakeholders, improved technological experience, and the inclusion of AI training in medical education programmes. Unmanaged risks and concerns could ultimately worsen the quality of the doctor–patient relationship.

From a prospective point of view, AI demonstrated its potential to possibly and directly improve this critical relationship [93]. Future pathways should be closely guided by the evidence that has emerged to avoid failures and prevent the creation of new limitations.

### 3.4. Doctor–Patient Relationship

Across studies, reductions in satisfaction were not uniformly reported across settings and, when present, mainly concerned satisfaction with the AI-supported care process rather than with the physician. These effects were more frequently observed in simulated or AI-forward scenarios in which relational aspects of care were perceived as diminished. Four main thematic domains directly related to the doctor–patient relationship emerged: attitudes towards AI, perceived risk, advantages/disadvantages, and emotional response to AI.

Attitudes toward AI influencing the relationship were examined in eight studies [87,90,91,94,100,101,102,107]. According to Al Fadeel et al. [87], in a study involving 105 physicians, 64% of participants reported a positive regard toward AI. For the majority of the sample, AI was not expected to affect the relationship, provided that human oversight was maintained, although differences emerged according to experience, sex, and years of service. Concerns were also highlighted by Ammann et al. [90] in a study including healthcare professionals, patients, and family members. While AI was associated with progress, professional identity, role perception, and human contact were described as irreplaceable. Similarly, Armero et al. [91], involving 349 parturients, reported that 69.2% of participants considered AI meaningful, although educational level emerged as a relevant variable. The presence of a human physician and the clinical relationship were considered irreducible, and concerns remained evident. Li et al. [94], in a study of 228 oncologists, reported additional worries related to misdiagnosis, inappropriate treatment, bias, and ethical issues, alongside a clear preference for human interaction. By contrast, Prabhath et al. [100], studying 250 medical students, found overwhelmingly positive attitudes toward AI, with participants stating that empathy and professional qualities should remain central to the doctor–patient relationship. Riedl et al. [101], including 1183 patients, confirmed the centrality of trust, while identifying privacy concerns, potential information disclosure, and qualified distrust as relevant patient considerations. These findings highlight the importance of an evidence-based foundation for AI implementation. Perceived risks represent a consistent concern for patients and, consequently, for the integration of AI into clinical practice. Rodler et al. [102] found that technological affinity and trust in AI were positively correlated, particularly when AI was controlled by physicians as an integrated clinical tool. AI operating independently was viewed as a source of concern among 446 cancer patients and was considered compatible with preserving the doctor–patient relationship only when used as an assisting tool. Similarly, Wu et al. [107], involving 541 patients, showed that physician-assisted AI and anthropomorphic framing were perceived as supportive of the relationship. Overall, attitudes toward AI were strongly influenced by technological familiarity, with AI primarily valued as an assistive resource. Autonomous AI systems were frequently associated with concerns and perceived reductions in care quality due to diminished human interaction.

Nine studies addressed advantages and disadvantages related to AI use [92,95,96,97,98,99,104,105,106]. Reported benefits included improved diagnostic capabilities and data management [92], as well as administrative efficiency [98]. However, concerns emerged regarding workforce implications and the erosion of human contact. Lombi and Rossero [95] noted that while decision-making authority was not perceived as compromised, radiologists reported tension related to professional epistemic authority, with potential relational implications. Individualised implementation strategies were suggested as a way to preserve the relationship. Mansour and Bick [96], involving 12 physicians, emphasised the importance of maintaining control over AI applications and developing awareness of their real capabilities and limits. Although AI was shown to improve treatment adherence [97], its use in documentation and formal tasks was viewed as particularly advantageous by patients [98]. Physicians expressed cautious optimism [99], stressing that training should accompany AI adoption and that communication, empathy, and trust must remain irreplaceable. Risks of reduced satisfaction and patient comfort in AI-supported care [104] were considered addressable through training and information literacy. Expectations should focus on integration rather than replacement of human contribution [105]. Umer et al. [106] similarly highlighted the importance of familiarity with AI grounded in awareness of its limitations and the enduring relevance of human contact.

Three studies specifically explored integration and alignment themes. Alkabi and Elsori [88] suggested that improved accessibility could strengthen the doctor–patient relationship when AI is meaningfully integrated into clinical workflows, despite infrastructural and cultural barriers. Allen [89] emphasised stakeholder alignment and technological integration as protective factors for the relationship. Schneider et al. [103] argued that responsibility and professional self-determination remain irreducible elements of medical practice, and that AI is best received when positioned as an integrative toolset. Only one study focused explicitly on emotional responses to AI within the doctor–patient relationship [93]. Findings suggested that AI-assisted assessment of affective dynamics in both patients and clinicians could enhance relational quality, representing a potential area of advancement. Further research in this direction appears warranted.

## 4. Discussion

This study aimed to identify and discuss evidence from published empirical studies on AI within the context of the doctor–patient relationship. Using a narrative review methodology, it was possible to capture and synthesise studies published over the last 10 years. All included studies provided information on AI use and its implications for the doctor–patient relationship. Through detailed analysis, three main thematic areas were highlighted: the use of AI from the patients’ perspective, the perspective of healthcare professionals, and the domain of academic education.

The decision to include exclusively empirical studies focused on the doctor–patient relationship was informed by existing literature showing that most contributions to date remain comprehensive or thematic. This approach allowed us to recognise how empirical evidence may inform future developments in the use of AI in the medical field. Consequently, this review underscores the value of existing evidence in guiding responsible AI applications in healthcare.

Analysis of the patient perspective offers critical feedback, given that patients are the ultimate recipients of healthcare services. According to other valuable studies, their point of view plays a central role in evaluating ongoing processes, particularly within broader health management contexts, where psychological factors such as illness perception, emotion regulation strategies, and perceived well-being significantly influence outcomes [108,109,110,111,112]. This underlines the importance of incorporating biopsychosocial dimensions into our understanding of patient experiences, even within the evolving role of AI in healthcare [113,114,115].

The results section refers to patients included in qualitative, quantitative, and prospective studies. On a qualitative level, fundamental factors such as trust, mistrust, and privacy concerns emerged as critical themes. Consistent with other recent studies, data protection was identified as one of the main challenges in AI use [116,117,118]. Ensuring the protection and security of patient information is therefore essential. As highlighted earlier, empathy, trust, and compassion remain particularly important for patients. Despite existing concerns, patients generally displayed positive attitudes toward AI, a finding supported by further prior studies directly focused on this topic [119]. Patients appeared to prefer AI as a supplementary tool rather than a complete replacement for human care.

Notably, patients’ knowledge of AI and familiarity with technology emerged as predictors of positive attitudes in line with other contributions [120,121,122]. Education and knowledge are directly involved in shaping patients’ perceptions and attitudes. Positive attitudes toward AI integration within standard clinical practices represent a clear advantage. However, concerns were also raised regarding the preservation of the patient-healthcare professional relationship, a preference for human interactions, and the potential for increased healthcare costs.

Nonetheless, positive attitudes clearly emerged from seven studies. These findings suggest actionable recommendations, including improved user training and system customisation to guide future AI development. Suggestions such as anthropomorphising AI interfaces may also help enhance acceptance. However, acceptance rates would not necessarily correspond to improvement of the doctor–patient relationship quality, so that data should be interpreted cautiously. Patients generally held favourable views of AI, with higher educational attainment and greater AI knowledge predicting increased acceptance as confirmable by additional contributions [122,123].

The convergence of qualitative and quantitative findings reinforces these main themes. As previously noted, examining limitations and future perspectives should always rely on evidence-based research. As a foundation for next steps, the studies suggest directions including the optimisation of AI–human collaboration and the establishment of standardised guidelines for ethical deployment.

Prospective studies and trials offered valuable longitudinal perspectives. In these contexts, AI was particularly effective in supporting administrative tasks, reducing errors, and mitigating risks related to unsustainable workloads consistently with available knowledge [124,125,126]. Enhanced treatment adherence and instant detection of potentially adverse factors in the clinical relationship were also highlighted as key benefits. These elements are fundamentally important in medicine, and AI is beginning to demonstrate a positive impact in these areas [127,128]. Again, patients’ education levels and knowledge of AI were important predictors of positive attitudes. Nevertheless, the preservation of the clinical relationship remained a top concern for patients.

From the patients’ perspective, the findings suggest that concerns about data protection, privacy, trust in AI systems, and the preservation of the doctor–patient relationship remain central. AI appears to be positively received when framed as an integrative tool within routine clinical practice rather than as a replacement for human care. Patients’ knowledge of AI emerges as a crucial moderating factor: higher educational levels are associated with greater familiarity and acceptance, whereas lower educational levels tend to correlate with mistrust. This highlights the importance of digital and information literacy as key determinants of patient attitudes. Across studies, the preservation of the human relationship consistently emerges as a fundamental priority in clinical settings. While existing concerns may introduce interpretative biases, targeted educational and information strategies could mitigate these effects and support more balanced perceptions. Overall, AI acceptance appears conditional upon its integration as a supportive instrument that safeguards the centrality of human interaction in care.

The perspective of healthcare professionals was especially valuable in corroborating patients’ views. Given their high level of education, these opinions were more specialised and nuanced. The analysis included qualitative, quantitative [60,129], and prospective contributions.

Qualitative contributions showed appreciation for AI integration with traditional practices. Consistent with recent research, this preference appears well founded [130,131,132]. However, identified risks included potential impacts on patient relationships, professional replacement, decision-making processes, and threats to epistemic and professional status. This cautious perspective was partly influenced by participants’ high educational levels, although years of professional experience and familiarity with technology also played important roles. Not all participants reported the same degree of familiarity with AI. According to further recent studies, familiarity with AI and related technologies significantly influences judgement [133,134]. This finding aligns with data from both patient and professional samples [123]. Among the recommendations to mitigate resistance and reduce risks was the direct involvement of clinicians in the development of AI tools.

Quantitative findings revealed concrete concerns about misdiagnosis, algorithmic bias, and stakeholder relationships. These risks are well known and debated in the literature, underscoring the need to account for their impact in future healthcare practice [135,136,137]. Overall, attitudes toward AI were positive, and inclusion of AI topics in academic curricula was viewed as important. The main recommendation, central to this study, involved maintaining a strong focus on the doctor–patient relationship. Strategies to strengthen patient-clinician alliance were identified as necessary. The risk of harming this relationship was carefully considered and recognised as central to the development of AI tools.

In order not to undermine patients’ trust, an additional aspect is the issue of explainability [95,101,102,103,104]. Explainability is the ability of AI systems to make their decision-making processes transparent and interpretable for human users. Explainability has emerged as a fundamental requirement to strengthen trust among both healthcare professionals and patients, since opaque or “black box” models can undermine clinical confidence and hinder shared decision-making. In the context of the doctor–patient relationship, explainable AI is not only a technical necessity but also an ethical imperative, as it enables clinicians to justify medical choices and communicate them effectively to patients. Conversely, limited explainability may exacerbate perceptions of bias, increase the risk of professional disempowerment, and compromise the therapeutic alliance. For these reasons, ensuring adequate levels of interpretability represents a priority for the safe and responsible integration of AI in healthcare practice.

From a prospective perspective, AI’s potential and future development pathways must consistently account for the role, impact, and quality of the doctor–patient relationship. Although only one prospective study was included, its findings reinforced the centrality of this relationship.

The final thematic domain concerned academic education. Two studies were included, both involving medical students. Data were heterogeneous. In one study, attitudes were highly positive, with students emphasising variables such as empathy, trust, emotional awareness, and ethics. The importance of considering the patient relationship was central. In the second study, familiarity with AI was lower and data were more mixed. This heterogeneity was mainly linked to limited technological familiarity and suggests the need for cautious interpretation. The quantitative findings were complex and nuanced. Nevertheless, there was general recognition of positive attitudes toward AI-driven progress. The study of AI from students’ perspectives is particularly interesting. Existing literature on this topic is diverse, often exploring attitudes, perspectives, and general outcomes [138,139,140]. The role of AI in medical education is gaining increasing attention, as portrayed by important works [141,142]. However, few studies have directly focused on its impact on the doctor–patient relationship. Emerging data must be contextualised, as they are not yet fully integrated into clinical dynamics. Nevertheless, younger participants may be more familiar with technology. Data should also be contextualised at an institutional level, since the economic development of institutions represents a fundamental factor.

Findings related to healthcare professionals reveal both areas of convergence with patient perspectives and domain-specific concerns. Similar to patients, clinicians predominantly supported the integration of AI into routine clinical practice rather than its substitution for human expertise. At a professional level, however, physicians expressed concerns about the potential erosion of medical skills, risks associated with decision-making processes, and threats to professional epistemic authority, with possible downstream effects on the doctor–patient relationship. A recurring recommendation across studies was the active involvement of healthcare professionals in the development and implementation of AI technologies, alongside the promotion of informed and responsible use. Levels of knowledge and familiarity with AI emerged as significant determinants of professional attitudes, with clear differences observed between clinicians with higher versus lower technological exposure. Strengthening information literacy and increasing professional participation in technological development were consistently framed as necessary steps toward safer and more effective integration.

AI use in medical settings raises important questions that need to be addressed [13], such as big data analytics, applicability and real advantages, integration, impact on patient–doctor relationship, and external informatic factors influencing AI. Limits and concerns remain relevant for both healthcare professionals and patients. However, rigorous scientific processes offer realistic pathways for their resolution. Evidence-based approaches provide essential data to support the development of sustainable solutions. Promoting empirical research and critical reflection on the influence of AI use on the doctor–patient relationship begins with analysing existing contributions. In this sense, the present review aimed to highlight existing evidence to encourage further study and to summarise the current state of the art.

## 5. Strengths and Limitations

This narrative review examined recent research on AI use within the context of the doctor–patient relationship. Several limitations should be acknowledged.

First, the limited number of included studies, the heterogeneity of study designs, the frequent use of non-validated instruments, and the high proportion of qualitative research may affect the interpretation of the findings. In particular, qualitative studies relying on ad hoc measures constrain comparability due to differences in methodological structure. Nevertheless, the consistency of themes observed across qualitative, quantitative, and prospective studies partially mitigates this limitation.

The small number of prospective studies and trials represents an additional constraint, although their results were generally aligned with those of quantitative investigations. As a narrative review, issues related to reproducibility and generalisability should also be considered. The included studies showed marked heterogeneity in AI type, outcomes, and design. Thirteen studies did not involve implemented AI systems and instead focused on perceptions, representations, and knowledge. Two studies evaluated simulated AI scenarios, while six examined real-world AI applications across diverse clinical settings, including primary care, oncology, radiology, and dentistry. Despite this methodological diversity, a notable convergence of findings emerged.

Despite these limitations, this review contributes to a more coherent understanding of recent developments in the field. To our knowledge, no previous review has directly and systematically examined the doctor–patient relationship in relation to AI use. The synthesis of current evidence, therefore, represents a meaningful step forward in clarifying an evolving area of research.

## 6. Conclusions

Patients’ representations of AI in relation to the doctor–patient relationship emerged as a central theme. Overall attitudes toward AI were generally positive, although strongly associated with educational level, knowledge, and technological familiarity. These patterns were consistently observed across studies. Alongside positive expectations and practical recommendations, patients expressed concerns about preserving the clinical relationship, highlighting the need to carefully consider factors shaping patient experience.

Healthcare professionals demonstrated similarly positive orientations toward AI, yet concerns persisted regarding the quality of the clinical relationship. Core professional values—including trust, empathy, and compassion—were repeatedly identified as essential. Despite typically high educational backgrounds, variations in years of experience and familiarity with AI technologies influenced professional responses. Future healthcare professionals echoed the importance of safeguarding these relational foundations.

More broadly, the centrality of the doctor–patient relationship received consistent emphasis. The limitations and concerns identified in this review align with existing literature, particularly regarding the shared priority of maintaining meaningful human connection in clinical care. These findings provide an evidence-based platform for future investigation and support the development of additional empirical research. Further studies should explore how AI implementation interacts with educational level, technological familiarity, and strategies designed to protect and strengthen the doctor–patient relationship.

## Figures and Tables

**Table 1:** Studies included in the narrative review main features.

Authors	Year	State	Research Design	Sample	AI Types and Applications	Measure	Findings
Al Fadeel et al. [87]	2021	Saudi Arabia	Cross-sectional quantitative research	105 physicians	No AI system implemented (AI discussed at a conceptual or perceptual level)	Ad hoc questionnaire	The study highlighted no significant differences related to sex, work category, and years of experience with reference to attitudes towards AI. In total, 64% of the involved physicians reported excellent attitudes towards AI.
Alkabi & Elsori [88]	2025	United Arab Emirates	Cross-sectional qualitative research	7 patients and 8 healthcare processionals	No AI system implemented (AI discussed at a conceptual or perceptual level)	Ad hoc semi-structured interview	The results suggest an enhancement of accessibility, convenience, and patient–doctor relationships. Integrating AI in typical clinical practices necessitates addressing contextual infrastructural and cultural hindrances.
Allen et al. [89]	2024	USA	Cross-sectional quantitative research	47 primary care physicians	No AI system implemented (AI discussed at a conceptual or perceptual level)	Specifically developed survey	According to the results, primary care physicians demonstrated technological concerns to be solved through a consistent alignment between primary care and stakeholders. Avoiding problem-solving strategies neglecting technological concerns would increase the rick for AI use failure. These issues represent fundamental features to be considered in order to preserve doctor–patient relationship.
Amann et al. [90]	2023	Switzerland, Germany, United Kingdom	Cross-sectional qualitative research	14 healthcare professionals, 14 stroke survivals, 6 family members	No AI system implemented (AI discussed at a conceptual or perceptual level)	Ad hoc semi-structured interview	Doctor–patient relationship concerns emerged among the patients, together with professional identity and role perception apprehension. Attitudes were mostly positive towards progress provided by AI in the clinical field. The opportunities provided by AI in the context should take into account possible incoming problems in order to avoid new limits. Emerged benefits were identified and appreciated by the subjects.
Armero et al. [91]	2022	USA	Cross-sectional quantitative research	349 parturients	No AI system implemented (AI discussed at a conceptual or perceptual level)	Specifically developed survey	The analysis provided for two different groups, respectively parturients preferring physician presence and those who preferred AI use. In total, 69.2% of patients recognised AI as meaningful and providing benefits. Parturients appreciating AI reported higher education rates. Despite most of the patients being optimistic about the advancement of AI, concerns referred to the preservation of the human physician–patient relationship emerged.
Ayad et al. [92]	2023	Germany, Netherlands	Cross-sectional quantitative research	265 dentistry patients	No AI system implemented (AI discussed at a conceptual or perceptual level)	Survey	Three main concerns emerged about the use of AI, regarding the impact of workforce needs, challenges to the doctor–patient relationship, and increased dental care costs. Advantages regarded improved diagnostic confidence, time reduction, and more personalised management.
Huang et al. [93]	2023	Taiwan	Prospective study	4 physicians, 348 dermatology patients, 326 clinical session videos	Multimodal analysis (computer vision, machine deep learning, emotion recognition)	Physician–patient satisfaction questionnaire, Facial Expression Recognition (ITRI)	Through the analysis of the clinical session material, emerged that doctors expressed more emotions than patients (anger, happiness, disgust, and sadness). Surprise was mostly showed by patients. The quality of a good doctor–patient relationship was testified by the presence of higher positive affectivity rates in the last part of the sessions. AI was used to assess detected affectivity.
Li et al. [94]	2024	China	Cross-sectional quantitative research	228 Chinese oncologists	No AI system implemented (AI discussed at a conceptual or perceptual level)	Survey	Concerns emerged regarding the use of AI and possible mislead diagnosis and treatment, data and algorithm bias, data security, and ethical issues. Sex differences were not significant and subjects with more technological experiences were more positive. Demographic and professional factors were not significantly correlated with the emerged concerns. Positive results emerged with reference to the preservation of doctor–patient relationship.
Lombi & Rossero [95]	2023	Italy	Cross-sectional qualitative research	12 radiologists	No AI system implemented (AI discussed at a conceptual or perceptual level)	In-depth interviews	AI emerged as not affecting radiologists’ decision-making process. However, professional and epistemic authority emerged as threatened by AI use. Results suggest the irreplaceability of knowledge extending beyond image interpretation. Fostering radiologists’ prestige through developing AI expertise was considered as a possible mediator. The implementation of the doctor–patient relationship was considered to be a possible target of AI use.
Mansour & Bick [96]	2024	United Arab Emirates	Cross-sectional qualitative research	12 physicians	Perceptions and knowledge of AI in the healthcare sector	Semi-structured interview	Need for physicians’ control of the applications, training and engaging in development phases emerged. Insurance, connection, and easy interpretability of AI outcomes represent a requirement. Patients should be involved in the use of AI and fully aware about the offered possibility in order to avoid negative consequences affecting the doctor–patient relationship.
Oh et al. [97]	2025	USA	Pilot randomised controlled trial	36 patients	Generative AI–conversational AI (Relational Chatbot, Natural Language Processing)	AI Chatbot, Linguistic Inquiry and Word Count (LIWC)	Constant engagement with AI provided higher rates of physical activity compared to the control group. Stronger interaction and greater feasibility were reported by subjects using the AI Chatbot.
Owens et al. [98]	2024	USA	Prospective observational study	288 patients (open label arm) and 304 patients (masked phase)	Natural Language Processing and Ambient Voice Technology	Patient–doctor Relationship Questionnaire-9 (PDRQ-9)	Patients of the open label arm reported more confidence and providers’ focus on them. Time typing was reduced and the encounter experienced as more personable. No differences in the patient–doctor questionnaire were found with reference to the subjects included in the masked phase. Patients consistently agreed with the use of ambient voice recognition and AI for documentation of primary care activities. No differences were found referring to the patient–physician relationship.
Pedro et al. [99]	2023	Portugal	Cross-sectional quantitative research	1013 physicians	No AI system implemented (AI discussed at a conceptual or perceptual level)	Survey	Medical community was optimistic regarding AI adoption for clinical practice. Disadvantages and challenges represent a concern, so that medical education should include AI tools training. Good communication and empathy would preserve doctor–patient relationship improving healthcare quality. This link should be taken into account in order to rise concerns about the AI replacement of clinical figures.
Prabhath et al. [100]	2025	India	Interventional study	250 medical students	No AI system implemented (AI discussed at a conceptual or perceptual level)	AI doctor–patient relationship tool and related validated questionnaire	The feedback was overwhelmingly positive since the sessions were described as significantly enhanced. Professional qualities and empathy were detected as reinforcing the doctor–patient relationship. Emotional and ethical dimensions related to medical practice were represented as fundamental. Visual hermeneutic approach demonstrated its potential cultivating empathy and fostering a deeper understanding of the relationship.
Riedl et al. [101]	2024	Austria, Germany	Cross-sectional quantitative research	1183 patients	Hypothetical/simulated AI decision-making system—no actual algorithm implemented	Vignettes on patient–doctor interaction	Significant variables such as trust, distrust, possibly perceived privacy invasion, information disclosure, treatment adherence, and satisfaction emerged as relevant for the patients. In particular, trust, distrust and privacy invasion predicted information disclosure, adherence, and satisfaction as interaction variables. Psychiatric practice differed consistently from other disciplines in the “human doctor with AI” condition. Trust and empathy represent fundamental values linked to doctor–patient relationship. Identifying their role and extent represent an added value for research.
Rodler et al. [102]	2024	Germany, USA	Prospective trial	466 prostate cancer patients	AI-based clinical decision support system	Specifically developed questionnaire	Affinity for technology was positively correlated with trust in AI. The highest rate of trust was referred to diagnosis made by AI controlled by the physician. AI-assisted physician was preferred over physician alone and AI alone. Trust in new developments and future diagnostic and therapeutic AI-based treatment emerged as linked to human–AI interaction. The doctor–patient relationship was preserved in the case of AI assisting physicians.
Schneider et al. [103]	2025	Germany	Cross-sectional qualitative study	18 patients	No AI system implemented (AI discussed at a conceptual or perceptual level)	Focus groups	AI-based clinical decision support systems (AI-CDSS) were perceived as a challenge both for current decision-making and future doctor–patient relationship. Trust, responsibility, and self-determination were represented as main figures linked to the medical profession. The results appeared as strongly influenced by the patients’ comprehension of such technologies. Information about new technologies should be part of educational path for both physicians and patients.
Sun et al. [104]	2023	China	Cross-sectional quantitative research	740 patients	Clinical decision-making support AI	AI trust questionnaire	According to the results deriving from the administration of a specific questionnaire (fairness theory type), physicians using AI can reduce patient’s satisfaction. Information provided by physicians can mitigate this effect. The study contributed to the literature pertaining to AI and fairness theory, formulating practical suggestions including the need for physicians to give patients clear information about AI methods in order to avoid satisfaction decrease and worsen the doctor–patient relationship.
Tanaka et al. [105]	2023	Japan	Cross-sectional qualitative research	9 physicians	No AI system implemented (AI discussed at a conceptual or perceptual level)	Focus groups and interviews	The study focused on doctors’ expectations regarding functions possibly replaced by AI, still expected of humans and concerns about the AI use in the medical field. Functions extended by AI expected as positive, with particular reference to massive data analysis. Responsibility, commitment, and relation with patients represent key points to be preserved and addressed.
Umer et al. [106]	2024	Pakistan	Cross-sectional quantitative research	351 medical doctors	No AI system implemented (AI discussed at a conceptual or perceptual level)	Survey	Considering the whole sample, only 21.3% of the participants reported familiarity with AI. Only 16% of subjects had good familiarity with AI. For the 47.9% of the participants, AI would not out-compete the physician in the important trait of professionalism. In total, 20.2% believed AI being diagnostically superior to human physicians; 61% were concerned about a complete trust towards AI for medical decisions; and 74.4% believed that AI should be used only for administrative tasks. Referring to replacement concerns, 46.2% of the subjects expressed low worry. In total, 64.1% of the future doctors demanded for AI integration in State Healthcare System. Finally, 58.1% of the participants suggested that AI would not mirror the patient–doctor relationship.
Wu et al. [107]	2021	China	Cross-sectional quantitative research	541 patients	Hypothetical/simulated AI decision-making system—no actual algorithm implemented	Questionnaire	Through 3 experiments, data showed how patients preferred human doctors with a certain treatment plan; the acceptance of the treatment plan was influenced by an interactional effect involving the treatment subject; experiences were mediated by treatment provider (human vs. AI) and treatment plan acceptance. Anthropomorphising stimuli would improve the human–computer interaction.

## Data Availability

No new data were created or analyzed in this study.
